# TbIRK is a signature sequence free potassium channel from *Trypanosoma brucei* locating to acidocalcisomes

**DOI:** 10.1038/s41598-017-00752-1

**Published:** 2017-04-06

**Authors:** Michael E. Steinmann, Remo S. Schmidt, Peter Bütikofer, Pascal Mäser, Erwin Sigel

**Affiliations:** 1 Institute of Biochemistry and Molecular Medicine, University of Bern, Bern, Switzerland; 2 Swiss Tropical and Public Health Institute, Basel, Switzerland; 3 University of Basel, Basel, Switzerland

## Abstract

Potassium channels from prokaryotes and eukaryotes are usually recognized by a typical amino acid sequence TXTGY(F)G representing the ionic selectivity filter. Using a screening approach with ion channel family profiles but without the above motif, we identified a gene in *Trypanosoma brucei* that exhibits homology to inward rectifying potassium channels. We report here cloning of this ion channel named TbIRK. The protein is localized to acidocalcisomes in procyclic and in bloodstream form parasites. Functional properties of this channel were established after expression in *Xenopus* oocytes. Currents recorded in potassium medium show inward rectification and little time dependence. Surprisingly, this channel retains selectivity for potassium ions over sodium ions >7, in spite of the lack of the classical selectivity filter. The sequence GGYVG was predicted in silico to replace this filter motif. Point mutations of the corresponding glycine residues confirmed this at the functional level. The channel is inhibited by caesium ions but remains unaffected by barium ions up to 10 mM. TbIRK is to our knowledge the first potassium channel in *T*. *brucei* that localizes to the acidocalcisomes, organelles involved in the storage of phosphates and the response to osmotic stress that occurs during the life cycle of trypanosomes.

## Introduction

The kinetoplastid parasites *Trypanosoma* spp. are the etiological agent of sleeping sickness prevalent in the Sub-Saharan part of Africa and Chagas disease which affects people in South-, Central and North America^1, 2^. Both diseases have severe impact on the health of the population as well as the economical situation of the endemic countries. Currently available treatments exhibit either poor efficiency or cause severe adverse effects. Thus the development of new and more effective drugs is urgent. Evaluation of new potential drug targets in the parasites may be the first step towards this goal. In this perspective ion channels are promising subjects based on their pharmacological accessability, sequence divergence from their human counterparts^3^ and lack of redundancy^4^.

So far only few ion channels from trypanosomatids have been described^5–7^, of which at least one was shown to be essential^7^. In addition, we have recently characterized two essential proteins in *T*. *brucei* that form a heteromeric potassium channel involved in the plasma membrane potential maintenance^8^. In the related parasite *Plasmodium berghei* it was demonstrated that knock-out of the potassium channel PbKch1 leads to impaired sexual reproduction of the mosquito stage^9^. These findings emphasize the role of ion channels as potential drug targets.

Potassium channels are a highly diverse family of membrane proteins present in virtually all organisms. Potassium channels are involved in various fundamental cellular functions such as maintenance of the plasma membrane potential, osmoregulation, sensing environmental changes and many more^10–12^. Based on their structural properties the potassium channels are divided into three main families, namely the 6TM (six transmembrane domain), 4TM and 2TM^13^. The latter is also known as the inward-rectifier potassium channel family. These proteins share the common feature of two transmembrane domains that flank a pore loop (P-domain) which forms the selectivity filter of the channel. The pore-forming subunits assemble in tetramers of homo- or heteromeric composition^14^. Heteromeric assembly does not only give rise to different functional properties but can also determine the localization of the channels^15, 16^. Modulation of rectifying potassium channels may be mediated by voltage changes, G-proteins, phosphatidylinositol-4,5-bisphosphate (PIP_2_), pH or ATP^17–20^. The majority of the inward rectifying potassium channels are blocked by Ba^2+^- and Cs^+^-ions^21, 22^.

Screening of the available *kinetoplastida* genomes with a core selectivity filter motif present in almost all potassium channels (XXGXGX) has earlier led to the identification several putative potassium channels belonging to the 6TM family^3^. Members of the 2TM or 4TM potassium channel families were not identified in the above study.

Using a different screening approach with ion channel family profiles, we identified a gene from *Trypanosoma brucei* (Tb927.11.12490) that exhibits homology to the inward rectifying potassium channels. Here this ion channel TbIRK is described. Although the protein is missing the classical K^+^-channel signature sequence we demonstrate that TbIRK is selective for potassium ions. Furthermore, an unusual selectivity filter was identified *in silico* and functionally confirmed by point mutations. TbIRK localizes to the acidocalcisomes in both procyclic and bloodstream form parasites. We suggest that TbIRK is involved in the functions ascribed to *Trypanosoma* acidocalcisomes such as polyphosphate (polyP) and cation storage^23^, Ca^2+^ signaling^7^, intracellular pH regulation^24^ and osmoregulation^25^.

## Results and Discussion

### Sequence analysis

Tb927.11.12490 was identified by an *in silico* screen with the hmmer-3.0b3 program^26^ of the predicted *T*. *brucei* proteome (v4.0 from *ftp*.*sanger*.*ac*.*uk*)^27^ using ion transporter family profiles from the Pfam database^28^. The gene Tb927.11.12490, in the following called TbIRK, was the only sequence that scored positive for the general inward rectifier potassium channel profile IRK (PF01007) with a full sequence E-value of 5.8e-6. The orthologues found in *T*. *cruzi* (TcCLB.511127.410) and *L*. *major* (LmjF.09.0480) exhibit at the protein level 41% (53% similarity) and 32% (42% similarity) identity, respectively (global Needleman-Wunsch alignment)^29^. The closest homologue found in the *Homo sapiens* genome is a G-protein-activated inward rectifier potassium channel (NP_002231.1) that exhibits 17% identity and 29% similarity at the amino acid level.

### A putative selectivity filter for TbIRK

**Figure 1:**
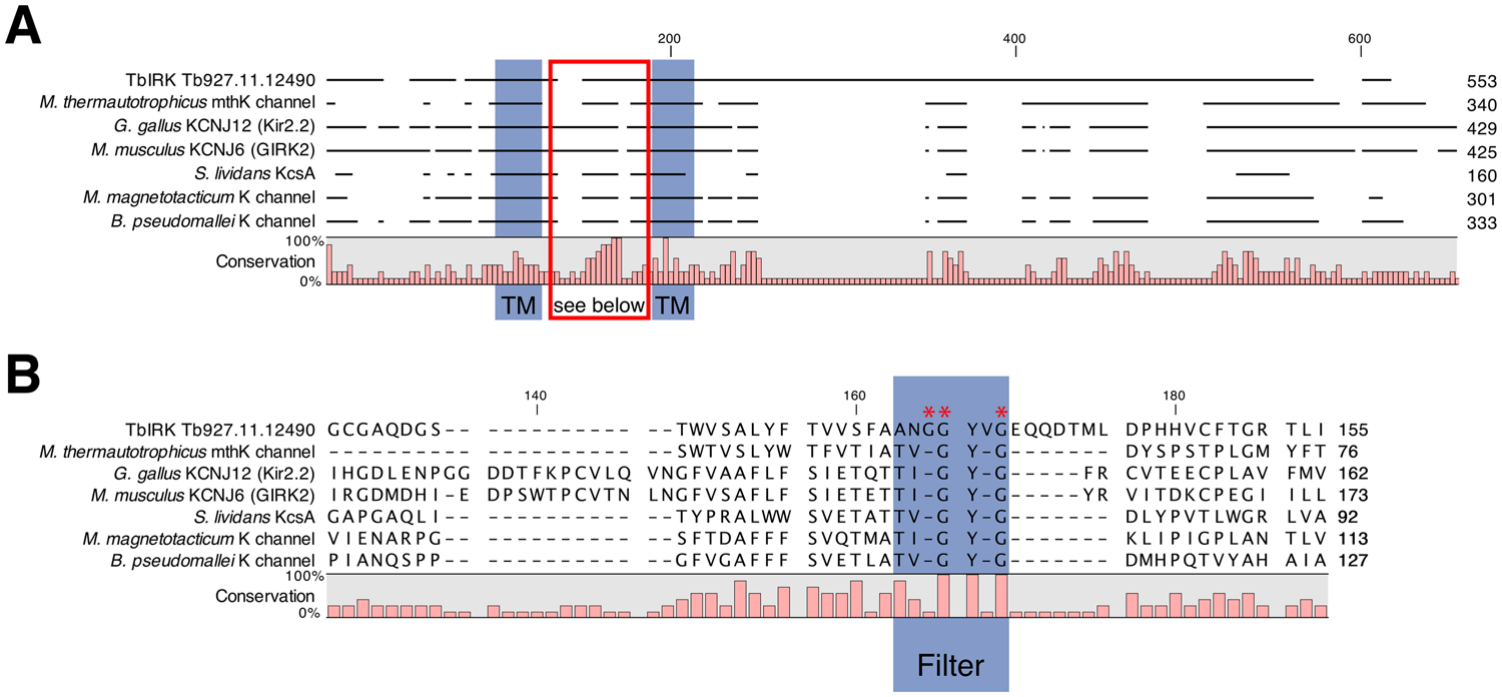
The unusual filter motif of TbIRK. (**A**) The protein sequence of TbIRK was aligned with representatives from six groups of similar proteins, as identified by HHBlits (Homology detection by iterative HMM-HMM comparison) within SWISS-MODEL^30^. Transmembrane domains unequivocally identified by different algorithms in all of the proteins are indicated in blue. The region between transmembrane domains is indicated by a red box. (**B**) Section indicated by the red box in (**A**), containing pore loop and filter motif, indicated in blue. Residues mutated within this study are indicated with a red asterisk.

Intriguingly, TbIRK does not contain the classical signature motif and we attempted to identify the alternative ion selectivity filter in this protein. Using BLAST against the TbIRK sequence, no reliable hits were found outside of the TriTryp-group, consisting primarily of *T*. *brucei spp*., *T*. *cruzi spp*. and *Leishmania sp*. We therefore looked for proteins with a similar arrangement of secondary structures. Using the homology model prediction algorithm of SWISS-MODEL^30^, we found eight groups of proteins (Fig. S1), similar to TbIRK. Of these eight groups two were eliminated, one for lack of coverage in the signature motif region and one for an interfering signal peptide, as predicted by Phobius^31^. Of each of the remaining six groups one protein with an available crystal structure was selected and compared to TbIRK (Fig. 1, Fig. S2). The two transmembrane domains in the studied proteins, including TbIRK are the only ones unequivocally predicted by the algorithms used. Unlike the other six groups, however, TbIRK does not contain the classical TxGYG motif but has the sequence GGYVG in this region. We hypothesized that this sequence might be the pore motif of TbIRK. By introducing point mutations into the putative selectivity filter, we could indeed alter the conductive properties of the channel (see below).

Other cases of proteins related to potassium channels with missing classical pore motifs include TMEM175^32^. This potassium ion selective ion channel posseses two groups of six putative transmembrane sequences. Other examples are the bacterial non-selective cation channels NirBacs, which have only been characterized by tracer flux in reconstituted liposomes^33^. The NirBacs have a classical P-loop flanked by transmembrane sequences, but the pore motif is drastically altered and the channels lose their selectivity for potassium ions.

### Electrophysiological characterization of TbIRK in *Xenopus* oocytes

**Figure 2:**
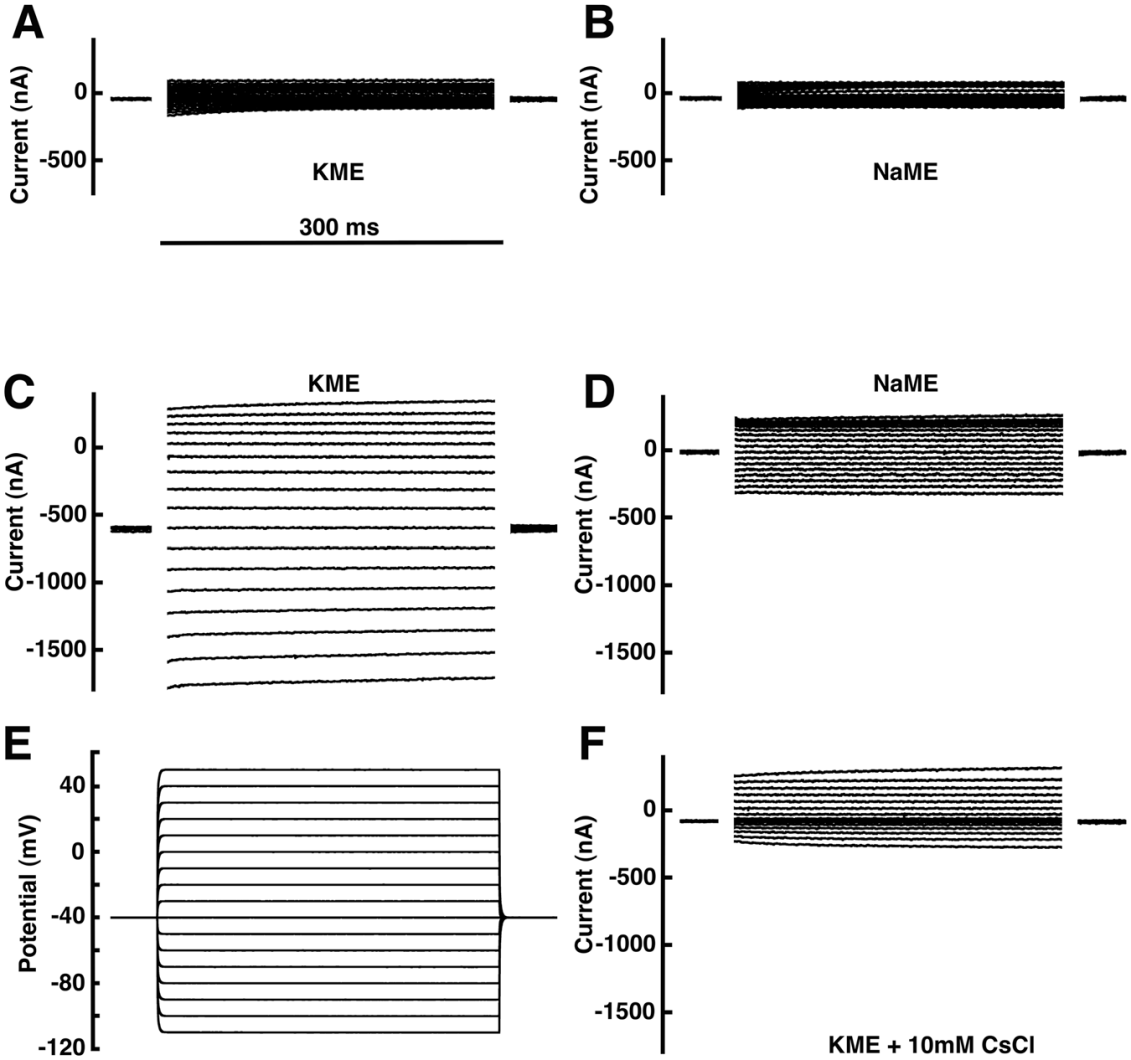
Electrophysiological characterization of TbIRK expressed in *X*. *laevis* oocytes. The 2-electrode voltage-clamp was used to study currents in oocytes injected with cRNA coding for TbIRK or with water. All recordings were done by applying the voltage-step protocol depicted in (**E**). The holding potential was −40 mV. With a frequency of 1 Hz voltage-steps of 300 ms duration were applied from −110 mV to 50 mV in 10 mV intervals. Currents recorded from a water-injected oocyte in potassium medium (KME) and sodium medium (NaME) are shown in (**A**) and (**B**). The corresponding representative current traces of a TbIRK-expressing oocyte in potassium medium and sodium medium (NaME) are shown in (**C**) and (**D**). (**F**) Recording of the same oocyte in potassium medium in presence of 10 mM CsCl.

**Figure 3:**
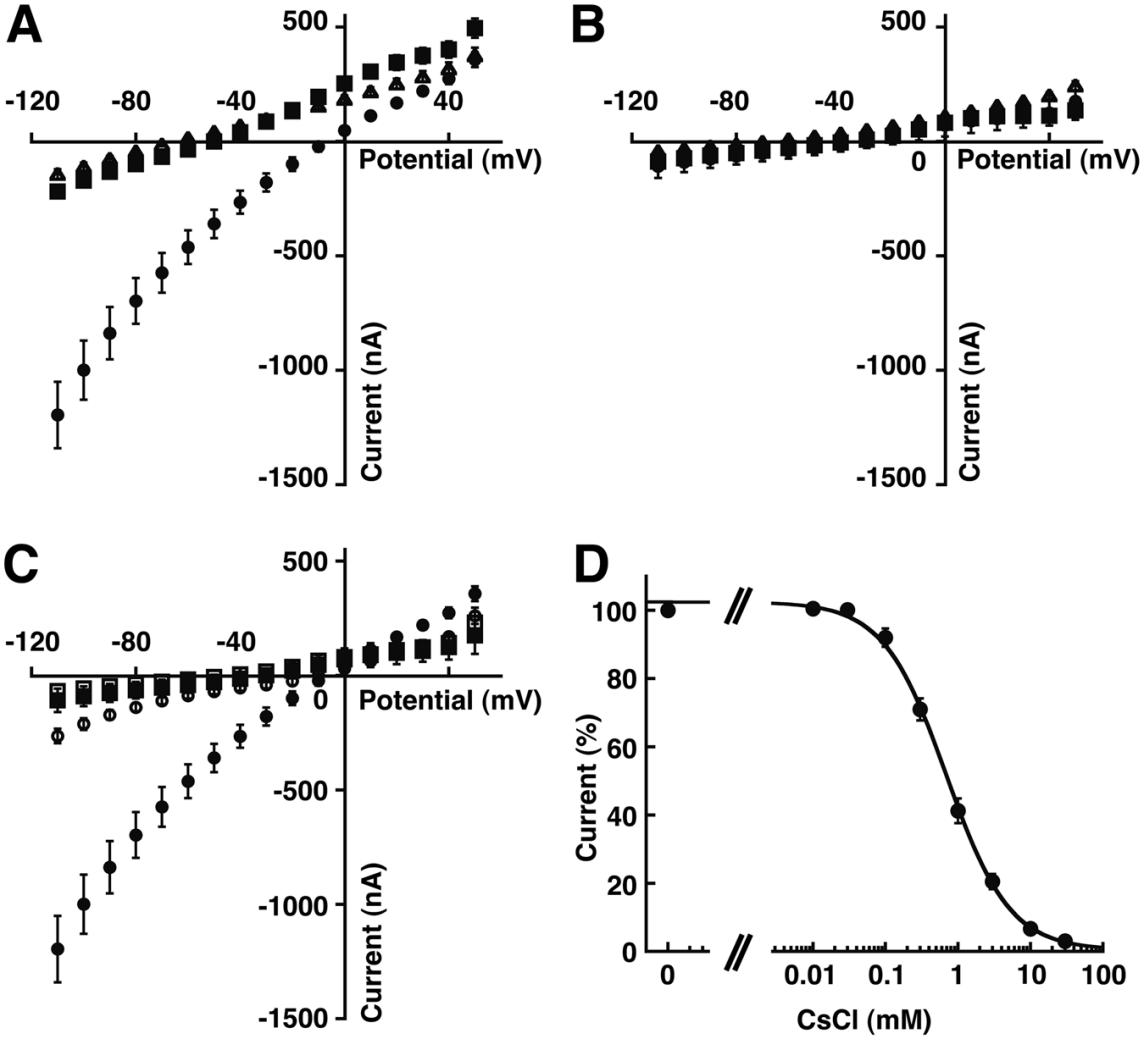
Current-voltage relationship. (**A**) Current-voltage relationship of TbIRK expressing oocytes in sodium medium (squares), potassium medium (circles) and *N*-methyl-*D*-glucamine medium (open triangels) (mean ± S.D.; n = 31 for sodium and potassium medium and n = 23 for *N*-methyl-*D*-glucamine medium). (**B**) The corresponding I/V relationship of water-injected oocytes in sodium medium (squares), potassium medium (circles) and *N*-methyl-*D*-glucamine medium (open triangels) (mean ± S.D.; n = 25 for sodium and potassium medium and n = 19 for *N*-methyl-*D*-glucamine medium). (**C**) Comparison of the I/V relationship of TbIRK expressing oocytes in potassium medium (circles) or sodium medium (squares) in presence (open symbols) or absence (closed symbols) of 10 mM CsCl (mean ± S.D.; n = 31 and 16, respectively for TbIRK-expressing oocytes and n = 25 an 14, respectively for water-injected control oocytes). (**D**) Inhibition by caesium was determined by changing from sodium medium to potassium medium containing different CsCl concentrations at −80 mV. The observed currents normalized to the response elicited by potassium medium in the absence of caesium. The inhibition curve was fitted with an IC_50_ of 0.74 ± 0.11 mM (mean ± S.E.M.; n = 7).

For the functional characterization of TbIRK, we successfully expressed the gene in *Xenopus* oocytes and applied electrophysiological techniques. This indicates that the channel protein, at least partially, assumes a plasma membrane localization in the *Xenopus* oocytes. For control purpose, representative current traces in potassium and sodium medium from an oocyte injected with water are shown in Fig. 2A and B, respectively. Three days after microinjection of cRNA coding for TbIRK, we found substantially larger inward currents at the holding potential of −40 mV when the perfusion medium was changed from sodium to potassium medium as compared to water-injected oocytes. For a more detailed characterization of the current mediated by TbIRK, we performed measurements applying a voltage-step protocol from −110 mV to +50 mV in intervals of 10 mV, starting from a holding potential of −40 mV (Fig. 2E). Representative current-traces are shown in Fig. 2C and D. In potassium medium, expression of TbIRK mediated large inward currents at negative potentials and small outward currents at positive potentials. During the pulses of 300 ms no time-dependent variation of the current was observed. Figure 3A and B show averaged current-voltage relationships of TbIRK expressing oocytes and water injected oocytes, respectively, in sodium, potassium and *N*-methyl-*D*-glucamine medium (NMDG). The amplitude of the latter currents in oocytes expressing TbIRK is higher than expected. This might be due to difference in oocytes leak current and/or health conditions as compared to water-injected oocytes.

Alternatively to the voltage-step protocol we applied a voltage ramp (Fig. S3). The two measurement strategies resulted in a similar current-voltage relationship, indicating the absence of time dependence of the TbIRK currents.

TbIRK-mediated currents in potassium medium were substantially reduced by caesium. To describe the inhibition of the channel by Cs^+^ in more detail, we measured the current induced by changing from sodium medium to potassium medium containing increasing concentrations of Cs^+^ at a holding potential of −80 mV. The inhibition of the current by Cs^+^ was fitted with an IC_50_ of 0.74 ± 0.11 mM (mean ± S.E.M.; n = 7; Fig. 3D). Endogenous conductances were not significantely affected by high (10 mM) concentrations of Cs^+^ (Fig. 3C). Ba^2+^ (10 mM), Cd^+^ (1 mM), tetraethylammonium (20 mM) and amantidine (100 μM), did not significantly influence the current. We are not aware of a potassium selective inward rectifier channel that is insensitive to 10 mM Ba^2+^. The activator for Slo2.2 channels^34^ bithionol (10 μM) as well as changes of the perfusion medium from pH 7.4 to pH 6.4 or 8.4 did not have a significant effect on the observed current.

Additionally, a set of nine compounds known to block members of the inward rectifier potassium channel family were tested (Table S1). Except for 400 μM rosiglitazone that showed an inhibition of 39 ± 7% (mean ± S.D.; n = 3) none of the compounds had a significant effect at the tested concentrations.

The reversal potential of oocytes expressing TbIRK was determined in medium containing Na^+^, K^+^ or NMDG as major cations. The reversal potential in sodium medium was −50 ± 2 mV (mean ± S.E.M.; n = 30), in potassium medium −10 ± 1 mV (mean ± S.E.M.; n = 30) and in NMDG medium −67 ± 2 mV (mean ± S.E.M.; n = 23). Using the Goldman-Hodgkin-Katz^35, 36^ equation, we estimated the relative permeability ratio p_K_/p_Na_ of the plasma membrane of TbIRK-expressing oocytes to be 7.3 (see also Materials and Methods). For water-injected control oocytes we found a relative permeability ratio p_K_/p_Na_ of the plasma membrane of about 1.3. As the currents through the plasma membrane are composed to a minor part from endogenous contributions and a major part mediated by TbIRK, the relative permeability ratio p_K_/p_Na_ of the channel is somewhat larger than 7.3. As inward rectifiers do not necessarily follow Hodkin-Huxley kinetics, we quantified currents observed at −110 mV in potassium (I_K_) and sodium medium (I_Na_) in TbIRK-expressing oocytes and subtracted those observed in control oocytes. I_K_/I_Na_ was 8.3. This value is similar to the one obtained using reversal potentials for the estimation of the selectivity.

### Point mutations in the predicted selectivity filter alter ion selectivity

The glycine residues in the predicted selectivity filter GGYVG at positions 131, 132 and 135 were mutated individually to alanine. These three point mutations had drastic effects on the survival rate of injected oocytes. While injection with water or mRNA coding for TbIRK resulted in little mortality, most oocytes injected with mRNA coding for one of the mutant channels showed damage, as indicated by pigmentation changes and low membrane resistance. Fewer than 10% of the oocytes expressing mutant channels could be analyzed using electrophysiological techniques. After 48 h in modified Barth medium 50–60% of the oocytes showed a white spot at the animal pole, presumably the nucleus floating to the surface membrane^37^. In this context it is worth mentioning that melittin, a pore forming peptide, leads to a similar phenomenon^38^. This was the first indication that the ion selectivity could be affected by the mutations.

**Figure 4:**
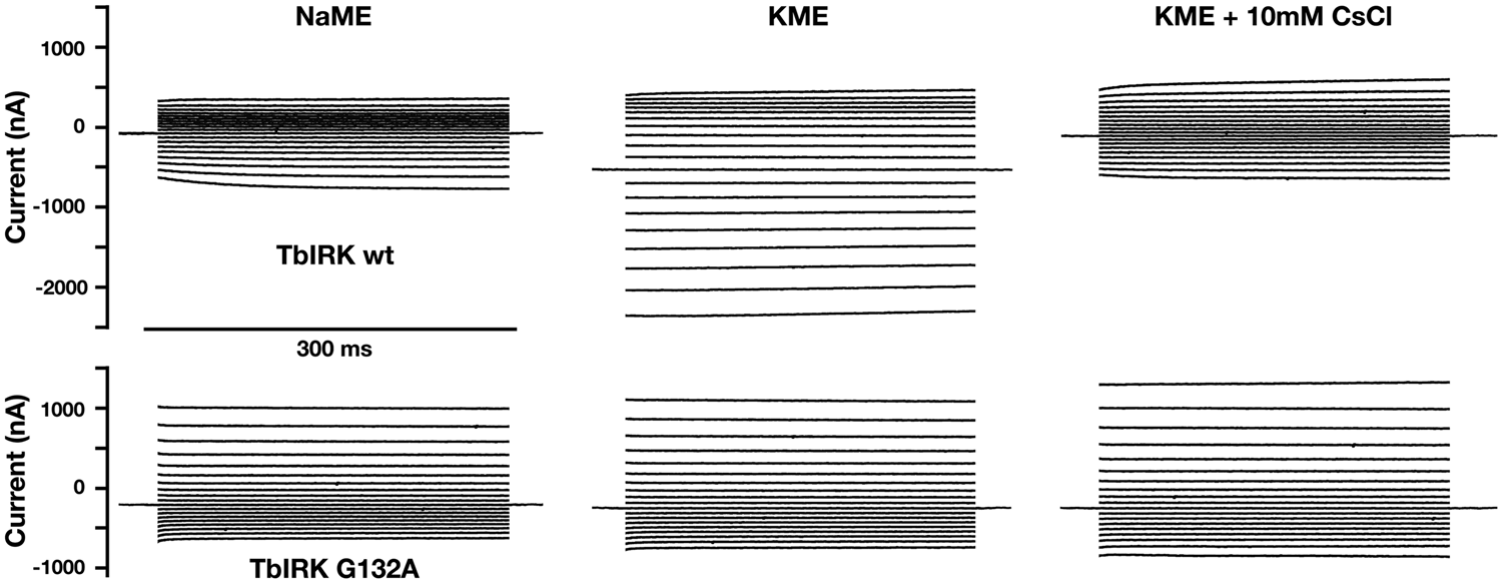
Loss of selectivity upon mutation of G132A located in the suspected selectivity filter. All recordings were done by applying the voltage-step protocol depicted in Fig. 2E. Representative current traces obtained from TbIRK-expressing oocytes in sodium medium (NaME), potassium medium (KME) and potassium medium supplemented with 10 mM CsCl are shown in the upper row. Recordings obtained with a oocyte expressing the G132A-mutant are shown in the lower row.

Surviving oocytes expressing mutant receptors were functionally characterized. One of the notable changes as compared to wild type receptors was a shift of the reversal potential determined in potassium medium (reversal potential of about −11 mV) close to the corresponding value determined in sodium medium (about −16 mV). Figure 4 shows original current traces in sodium and potassium medium and potassium medium supplied with 10 mM CsCl recorded from oocytes expressing wild type and mutant G132A TbIRK obtained using a voltage-step protocol depicted in Fig. 2E. The corresponding averaged current-voltage relationships of the mutant channel are shown in Fig. S4. Interestingly and for reasons that are far from clear, inward rectification was converted into an outward rectification upon mutation. Current amplitudes determined at −80 mV in sodium medium relative to those in potassium medium amounted to 22 ± 3% (mean ± S.E.M., n = 10) and 73 ± 7% (mean ± S.E.M., n = 6) in wild type and mutant G132A, respectively. Inhibition by 10 mM Cs^+^ was determined similarly for wild type channels and the mutant G132A. Residual current amounted to 23 ± 3% (mean ± S.E.M., n = 10) and 81 ± 11% (mean ± S.E.M., n = 6) in wild type and mutant channels, respectively. As compared to wild type channels, the mutant showed enhanced conduction of Na^+^ and decreased inhibition by Cs^+^. Similar observations were made with the mutants G131A and G135A, but the number of oocytes measured was too small to reliably report quantitative values. We conclude that at least the mutation G132A has a large impact on the ion selectivity of the channel, reducing the selectivity for potassium over sodium ions. Moreover, this mutation affects the recification properties of the channel.

### Localization of TbIRK in procyclic and bloodstream form parasites

**Figure 5:**
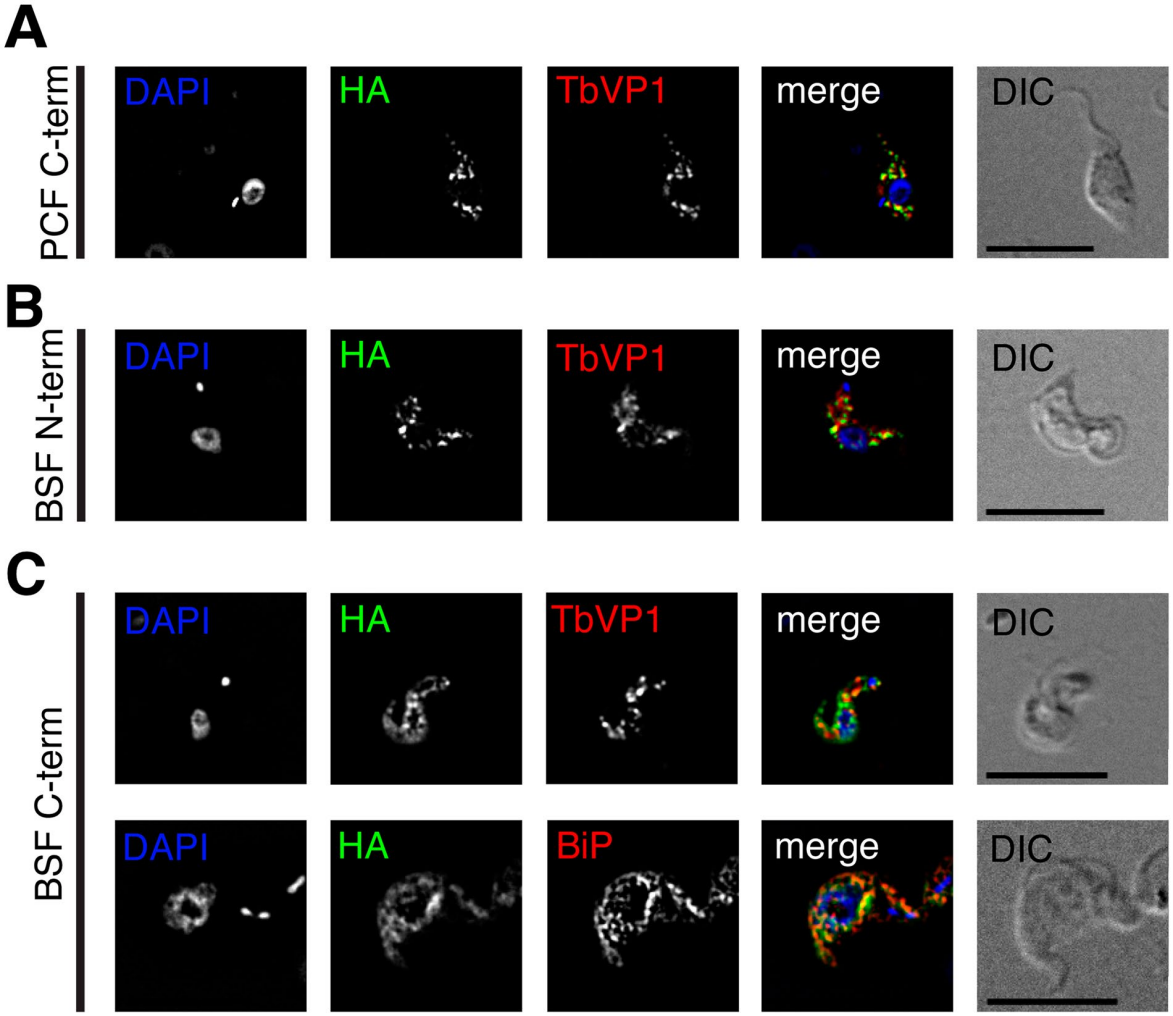
TbIRK co-localizes with a marker for acidocalcisomes. Co-localization of hemagglutinin (HA)-tagged TbIRK (green) with the acidocalcisomal marker TbVP1 (red) for C-terminally tagged TbIRK in procyclic form parasites (PCF, **A**) and N-terminally tagged TbIRK in bloodstream form parasites (BSF, **B**). (**C**) With the HA-tag at the C-terminus of TbIRK, its signal (green) partially co-localizes with TbVP1 (red, top) and partially with the ER marker BiP (red, bottom). Cells were counterstained with DAPI, shown in blue, visualizing the nuclear and kinetoplast DNA. DIC, differential interference contrast. Scale bars indicate 10 µm.

To determine the localization of the protein in the parasites we generated stably transfected cell lines expressing tetracycline-inducible C- and N-terminal hemagglutinin (HA)-tagged versions of TbIRK. Each HA-construct was verified by Western blot (Fig. S5). Cells were analyzed by immunofluorescence microscopy 24 h post-induction for procyclic and 48 h post-induction for bloodstream form parasites. TbVP1^24^, a vacuolar pyrophosphatase in *T*. *brucei*, was used as acidocalcisome marker. C-terminally tagged TbIRK in procyclic form parasites (Fig. 5A) and N-terminally tagged TbIRK in bloodstream form parasites (Fig. 5B) localized to acidocalcisomes. In procyclic form parasites N-terminal tagging did not yield a visible signal. In bloodstream form parasites C-terminally tagged versions partially localized to acidocacisomes and partially to the endoplasmic reticulum (Fig. 5C). It has previously been shown that a tagged protein may be retained in the endoplasmic reticulum on its way to the final destination^39^.

Pearson’s correlation coefficients (PCC) characterizing co-localization of TbVP1 with TbIRK for three biological replicates of C-terminally HA-tagged TbIRK in procyclic form parasites were 0.71 ± 0.08 (n = 13), 0.75 ± 0.08 (n = 12), 0.78 ± 0.04 (n = 10) (mean ± S.D., n = number of cells). In bloodstream form parasites, PCC for two biological replicates of N-terminally HA-tagged TbIRK were 0.70 ± 0.04 (n = 11), 0.69 ± 0.07 (n = 3) (mean ± S.D., n = number of cells). For the C-terminal clone partially co-localizing, it was 0.54 ± 0.08 (n = 7). PCC in two biological replicates for the ER-marker BiP^40^ and C-terminally HA-tagged TbIRK in BSF was 0.67 ± 0.04 (n = 3) and 0.67 ± 0.04 (n = 2).

We were thus able to localize HA-tagged TbIRK to acidocalcisomes. To our knowledge, this is the first time a potassium channel has been localized to the acidocalcisomes. The role of potassium in these organelles is not clear yet. Reported K^+^-concentrations in acidocalcisomes of trypanosomatids are dependent on the species and cultivation conditions^23, 41^. Surprisingly, the protein was not found in a recent proteomic analysis conducted on *T*. *brucei* acidocalcisome-rich fractions^42^.

Acidocalcisomes are found in eukaryotic as well as prokaryotic species. In trypanosomatids the acidocalcisomes have been studied in most detail. The cellular functions of the acidocalcisomes described so far are polyphosphate (polyP) and cation storage^23^, Ca^2+^ signaling^7^, intracellular pH regulation^24^ and osmoregulation^25^. Li *et al*.^25^ have investigated changes in the global gene expression following hyperosmotic stress in *T*. *cruzi*. The mRNA coding for the TbIRK ortholog in *T*. *cruzi* (TcCLB.509029.20) was down-regulated only transiently by 22%^25^. This observation indicates that TbIRK ortholog in *T*. *cruzi* may only play a minor role in osmoregulation.

### RNAi-mediated downregulation of TbIRK

**Figure 6:**
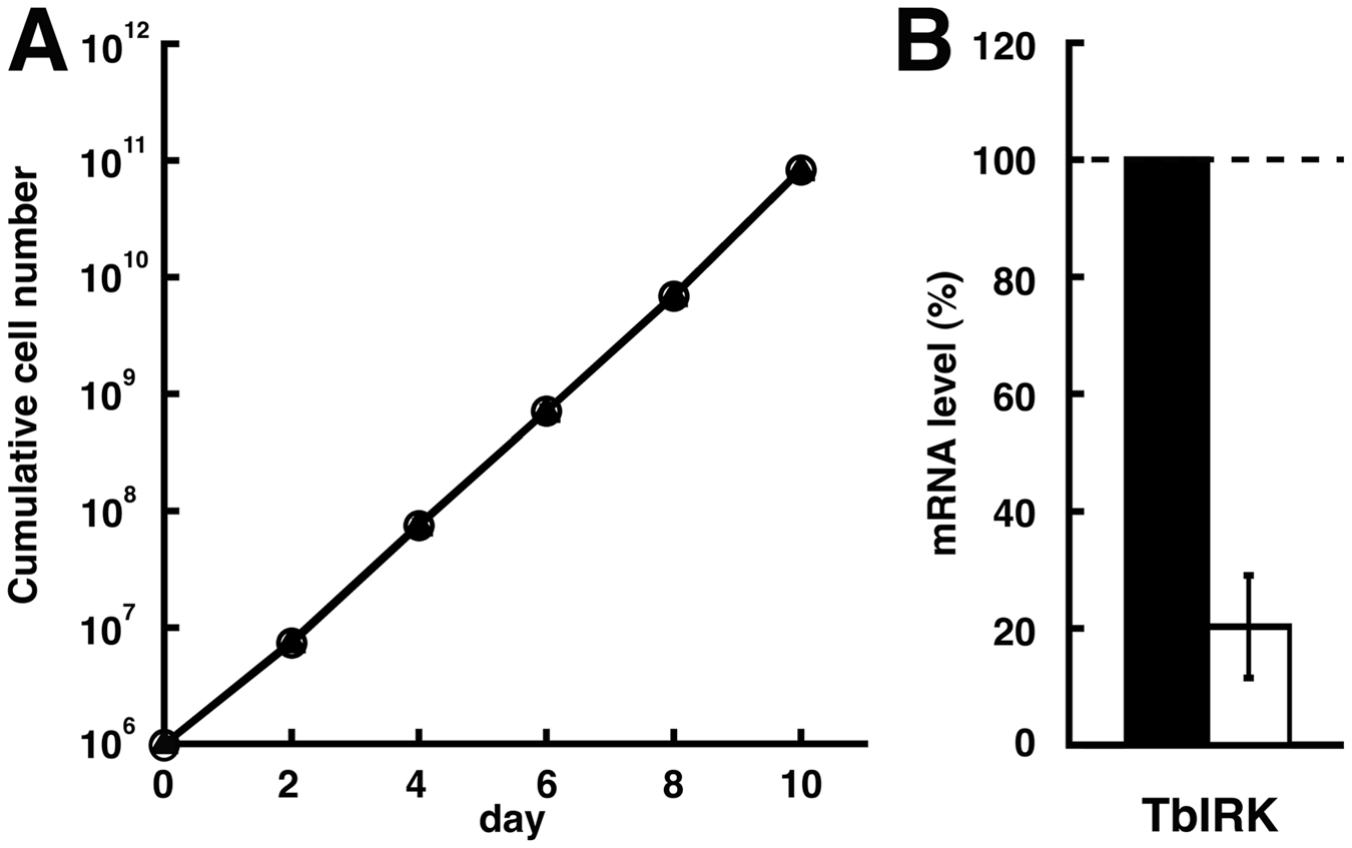
RNAi-mediated downregulation of TbIRK in procyclic form parasites. (**A**) Growth of non-induced (open circles) and induced (triangles) was monitored over 10 days (mean ± S.D., n = 3; error bars are smaller than the symbols). Induction of RNAi against TbIRK did not result in a growth phenotype. Changes in target mRNA levels were confirmed by qPCR (**B**). The black bar represents the mRNA level of non-induced cells and the white bar represents the mRNA level of TbIRK in induced cells. TbIRK mRNA was downregulated by 80 ± 9% (mean ± S.D., n = 3).

To investigate whether the protein is essential, we down-regulated its expression using inducible RNAi in both procyclic and bloodstream form parasites. Induction of RNAi by tetracycline did not affect the growth of procyclic form parasites (Fig. 6). To verify the efficiency of the RNAi constructs, we isolated total RNA from the tested clone 48 h after induction. The change in mRNA level of TbIRK was determined by qPCR. We found a down-regulation of the TbIRK mRNA of 80 ± 5% (n = 3; mean ± S.D.) in procyclic form parasites. For bloodstream form parasites, the target mRNA was not efficiently down-regulated (<50%, n = 3).

### Summary

In summary, we describe here an ion channel in *T*. *brucei*, TbIRK, with a moderate selectivity for potassium ions, but lacking the classical signature sequence forming the selectivity filter. Except for its insensitivity to Ba^2+^, the channel is structurally and functionally reminicent of inward rectifier potassium channels. TbIRK localizes to acidocalcisomes. It remains to be described how this potassium channel is involved in the acidocalcisome functions including polyphosphate (polyP) and cation storage^23^, Ca^2+^ signaling^7^, intracellular pH regulation^24^ and osmoregulation^25^.

## Methods

### Expression in *Xenopus* oocytes

Animal experiments were carried out in strict accordance to the Swiss ethical guidelines, and have been approved by the local committee of the Canton Bern Kantonstierarzt, Kantonaler Veterinärdienst Bern (BE85/15). Surgery of *Xenopus laevis* to obtain the oocytes was done under anesthesia, and all efforts were made to diminish animal suffering. Oocytes were prepared, injected and defollicated as described previously^43, 44^. Polyadenylated cRNA coding for Tb927.11.12490 (TbIRK) was prepared *in vitro* with the mMESSAGE mMACHINE kit (Ambion, Austin, TX, USA). The point mutations G131A, G132A and G135A were prepared using the QuikChange^TM^ mutagenesis kit (Stratagene, Agilent Technologies, Basel, Switzerland). Oocytes were injected with 50 nl of solution containing cRNA coding for wild type TbIRK (0.4 µg/µl), mutant TbIRK (0.3 µg/µl) or water and then incubated in modified Barth’s solution (10 mM HEPES, pH 7.5, 88 mM NaCl, 1 mM KCl, 2.4 mM NaHCO_3_, 0.82 mM MgSO_4_, 0.34 mM Ca(NO_3_)_2_, 0.41 mM CaCl_2_, 100 units/ml penicillin, 100 µg/ml streptomycin) at 18 °C for 3 days before measurements.

### Functional characterization in *Xenopus* oocytes

Electrophysiological experiments were performed using an Oocyte Clamp OC-725 (Warner Instrument Corp., Hamden, USA) two-electrode voltage clamp amplifier. Currents were digitized at 5 kHz with MacLab/200 (AD Instruments, Spechbach, Germany).

The holding potential was −40 mV, except for measurements of the concentration dependent inhibition by caesium chloride. With a frequency of 1 Hz discrete voltage steps of 300 ms duration to potentials ranging from −110 mV to +50 mV in steps of 10 mV were applied. The perfusion medium (NaME) contained 90 mM NaCl, 1 mM KCl, 1 mM MgCl_2_, 1 mM CaCl_2_ and 5 mM Na-HEPES (pH 7.4). The perfusion solution (6 ml/min) was applied through a glass capillary with an inner diameter of 1.35 mm, the mouth of which was placed about 0.5 mm from the surface of the oocyte^45^. When potassium was used as the major cation the medium (KME) contained 91 mM KCl, 1 mM MgCl_2_, 1 mM CaCl_2_ and 5 mM K-HEPES (pH 7.4). For determination of ion-selectivity of the channel an additional medium with N-methyl-D-glucamine (NMDG) as major cation was used. This medium contained 90 mM NMDG, 1 mM KCl, 1 mM MgCl_2_, 1 mM CaCl_2_ and 5 mM K-HEPES (pH 7.4). The K_ir_ channel blocker explorer kit (EK-112) was purchased from alomone labs (Jerusalem BioPark (JBP), Jerusalem 9104201, Israel).

For the determination of ion selectivity of the plasma membrane of oocytes expressing TbIRK, the Goldman-Hodgkin-Katz equation^35, 36^ was used as follows: E_Na_ = −50 mV = 58.8 mV * log((p_Na_*[Na_out_] + p_K_*[K_out_] + p_Cl_*[Cl_in_])/x), [Na_out_] = 92.5 mM, [K_out_] = 1 mM; E_K_ = −10 mV = 58.8 mV * log((p_Na_*[Na_out_] + p_K_*[K_out_] + p_Cl_*[Cl_in_])/x), [Na_out_] = 0 mM, [K_out_] = 93.5 mM; E_NMDG_ = −67 mV = 58.8 mV * log((p_Na_*[Na_out_] + p_K_*[K_out_] + p_NMDG_*[NMDG_out_] + p_Cl_*[Cl_in_])/x), [Na_out_] = 0 mM, [K_out_] = 3.5 mM, [NMDG_out_] = 90 mM. [Cl_in_] was determined as 30 mM from the reversal potential of oocytes expressing GABA activated recombinant chloride selective GABA_A_ channels. p_NMDG_ was assumed to be negligible. p_K_/p_Na_ can then be calculated to be 7.3. E_Na_, E_K_ and E_NMDG_ were determined experimentally. In water-injected control oocytes E_Na_, E_K_ and E_NMDG_ were in average −35 mV, −30 mV and −67 mV, respectively. p_K_/p_Na_ can be calculated to be 1.3 in this case. As these small endogenous conductances are present in all measured oocytes the true ion selectivity of the K^+^-channel is somewhat higher. Alternatively, we used the approach described by Heginbotham *et al*.^46^ for the estimation of p_K_/p_Na_ of oocytes expressing TbIRK and obtained a value of 10.2.

### RNAi-mediated gene silencing

Expression of Tb927.11.12490 was down-regulated by RNAi using a stem loop construct containing a puromycin resistance gene. Selection of the gene sequences for RNAi was done with RNAit, a prediction algorithm designed to prevent potential cross-talk and hence off-target effects^47^.

The chosen 411bp-long fragment spanning nucleotides 869–1279 of Tb927.11.12490 was amplified by PCR with the forward primer GCCCAAGCTTGGATCCCCGTTCAGACAGAAACCCAT and the reverse primer CTGCTCTAGACTCGAGGGTTAATCGTGTGCGTGATG and cloned into pALC14^48^ (a kind gift of André Schneider, University of Bern) allowing for tetracycline-inducible expression of a hairpin RNA under an rRNA promoter. Before transfection of *T*. *brucei* cells, plasmid DNA was linearized with *Not*I followed by a phenol/chloroform-extraction and subsequent precipitation.

Genomic DNA of selected clones was isolated and tested by PCR for the correct integration of the RNAi constructs.

### Stable transfection of trypanosomes and selection of clones

*T*. *brucei* 29–13 procyclic forms^49^ were cultured to mid-log phase (0.5–0.8 × 10^7^ cells/ml) in SDM-79 (BioConcept Ltd., Allschwil, Switzerland) supplemented with 10% (v/v) fetal bovine serum (LuBioScience GmbH, Lucerne, Switzerland) at 27 °C. 4–5 × 10^7^ cells were harvested by centrifugation at 1250 × *g* for 10 min, washed once in buffer (132 mM NaCl, 8 mM KCl, 8 mM Na_2_HPO_4_, 1.5 mM KH_2_PO_4_, 0.5 mM magnesium acetate, 0.09 mM calcium acetate, pH 7.0), resuspended in 450 μl of the same buffer, and mixed with 12 μg of linearized plasmid. Electroporation was performed with a BTX Electroporation 600 System (Axon Lab, Baden, Switzerland) with one pulse (1.5 kV charging voltage, 2.5 kV resistance, 25 mF capacitance timing, and 186 ohm resistance timing) using a 0.2-cm pulse cuvette (Bio-Rad Laboratories AG, Cressier, Switzerland). Electroporated cells were immediately inoculated in 10 ml of SDM-79 containing 10% heat-inactivated FBS. Clones were obtained by limited dilution in 24-well plates in SDM-79, containing 20% conditioned medium, in the presence of 2 μg/ml puromycin (Sigma-Aldrich, Buchs SG, Switzerland) for selection. Antibiotic-resistant clones were tested for the presence of the introduced constructs by PCR. RNAi or overexpression of tagged protein was induced by addition of 1 μg/ml tetracycline (Sigma-Aldrich) to parasite cultures.

### RNA isolation and qRT-PCR

Induced and non-induced RNAi cultures were harvested at various time points and total RNA was extracted according to the manufacturers protocol using the Promega SV total RNA extraction Kit (Promega Corporation, Madison WI, USA). RNA concentration and purity were measured with a spectrophotometer (Thermo Scientific NanoDrop™ 1000, Thermo Scientific, Waltham, MA, USA) and 5–10 µg total RNA of each time point was subjected to a second DNAseI treatment (Roche, Basel, Switzerland). Afterwards the RNA was purified by a phenol/chloroform-extraction followed by precipitation. RNA concentration was again determined with a spectrophotometer (NanoDrop™ 1000, Thermo Scientific) and 2 µg total RNA was used for a reverse transcription with random primers using the High Capacity cDNA Reverse Transcription Kit (Applied Biosystems, Life Technologies Ltd., Paisley, UK). The resulting cDNA was diluted to the desired concentrations and was analyzed by quantitative PCR using the Fast SYBR Green Kit (Applied Biosystems, Life Technologies Ltd., Paisley, UK) according to the manufacturer’s instruction. As validated internal control the expression levels of Tb11.01.1950 were measured in parallel^50^. The specific primers for the target were designed with the Primer3Plus-tool^51^. The forward and reverse primers used to amplify a 164 bp long product at position 733–896 of Tb927.11.12490 were CGCAAGAGGCGTAAGAGTTC and CTATGCGGATGGGTTTCTGT, respectively. For Tb11.01.1950 we used the primers that were published by Brenndörfer and Boshart^50^. Each sample was analyzed in triplicates and the average threshold cycle (Ct) was calculated for each sample. The relative expression levels of the target genes were determined by the 2^−∆∆CT^ method^52^.

### Immunofluorescence and microscopy

To generate overexpressable HA-tagged TbIRK we used the pALC14^48^ vector for procyclic form parasites and pMS14v5^53^ for bloodstream form parasites as described before^54^. In both cases C-terminal as well as N-terminal constructs were prepared. All constructs were linearized with *Not*I, extracted with phenol/chloroform and precipitated prior to transfection. For immunolocalization, the cells were cultured in presence of tetracycline for 24 h (procyclic forms) or 48 h (bloodstream forms) to induce expression of the tagged proteins. The cells were harvested by centrifugation at 800× g for 5 min, washed with cold phosphate buffered saline (PBS) and then spread on Superfrost Plus Microscope Slides (Thermo Scientific, Braunschweig, Germany). Preparation of the slides for microscopy was done as described before^55^.

The following primary antibodies were used; mouse monoclonal anti-HA (Covance, Princeton, NJ, USA), rabbit anti-BiP (a kind gift of J.D. Bangs, University of Buffalo, Buffalo, NY, USA) and rabbit-anti TbVP1 (a kind gift of R. Docampo, University of Georgia, Athens, GA, USA) at dilutions of 1:250, 1:1000 and 1:1000, respectively. The corresponding secondary fluorophore-conjugated goat anti-mouse Alexa Fluor 488 or goat anti-rabbit Alexa Fluor 594 (Invitrogen) antibodies were added at dilutions of 1:1000.

Fluorescence microscopy was performed on a Leica DMI6000 B microscope (Leica Microsystems). Deconvolution and analysis of the images was done with the Leica Application Suite X software provided by the manufacturer.

Image postprocessing was carried out in Icy^56^ (available at http://icy.bioimageanalysis.org/). Calculation of Pearson correlation coefficients was done using the Icy plugin Colocalization Studio^57^. We calculated 3D co-localization of green and red pixels within regions of interest enclosing a single cell in 3D-deconvolved pictures.

## Electronic supplementary material

Supplementary Information
