# Supplementary material for: TbIRK is a signature sequence free potassium channel from Trypanosoma brucei locating to acidocalcisomes
Source: Sci Rep. 2017 Apr 6;7:656. doi: 10.1038/s41598-017-00752-1 (PMC5429665; doi:10.1038/s41598-017-00752-1)
Supplement: Supplementary file 1 — Supplementary Information [file 41598_2017_752_MOESM1_ESM.pdf]

TbIRK is a signature sequence free potassium channel from *Trypanosoma brucei* locating to acidocalcisomes

Michael E. Steinmann[*], Remo S. Schmidt[†], Peter Bütikofer[*], Pascal Mäser[†,§], Erwin Sigel[*,1]

Figure S1. SWISS-MODEL cluster view of sequence similarity between proteins. After performing a similarity search, SWISS-MODEL groups template sequences and clusters them according to their relative similarity. Thus, XY coordinates are meaningless, only distances between the groups are relevant. One member of each group is listed on the right. Groups 1 and 4 were left out for further analysis, as group 1 did not align well in the N-terminal region, and group 4 has a predicted signal peptide on the N-terminus.

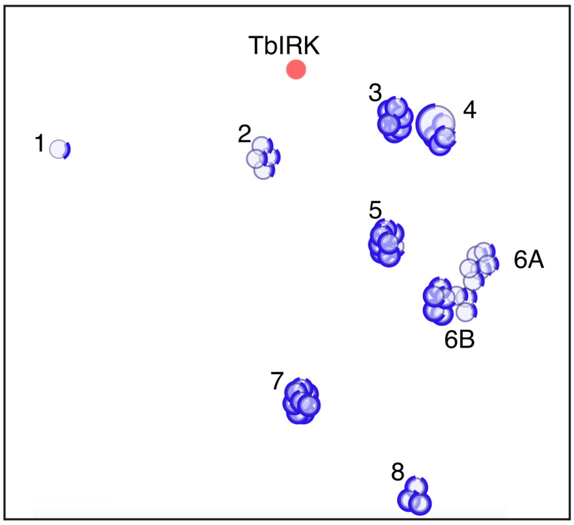

TbIRK: *T. brucei* Tb927.11.12490
1: *O. cuniculus* TRPV2
2: *M. thermoautotrophicus* mthK
3: *G. gallus* KCNJ12 (Kir2.2)
4: *M. musculus* Kir2.1
5: *M. musculus* KCNJ6 (GIRK2)
6A: *S. lividans* KcsA
6B: *M. musculus* Kir3.1
7: *M. magnetotacticum* K channel
8: *B. pseudomallei* K channel

Figure S2. Full-length alignment of TbIRK with representatives of six groups of similar proteins. Representatives of six groups of similar proteins identified by HHblits within SWISS-MODEL were aligned to TbIRK using the MUSCLE algorithm. The alignment at the filter pore (position 163-170) was manually curated to better show the alignment between filter motifs. Data was visualized in CLC Sequence Viewer.

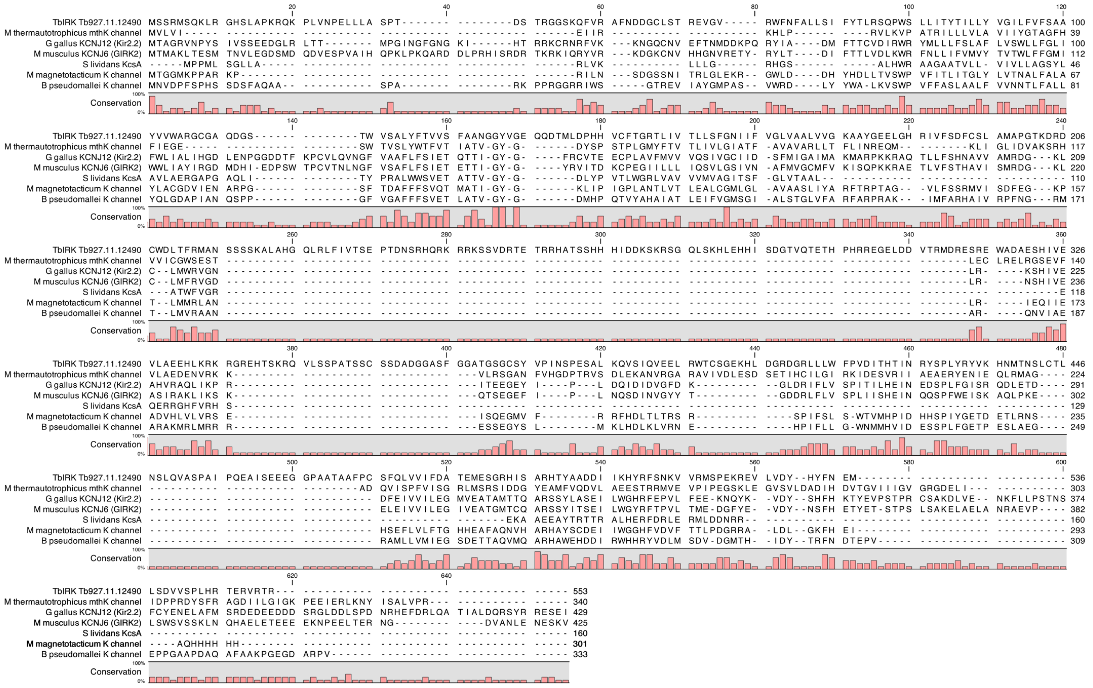

Figure S3. Representative current traces of a TbIRK-expressing oocyte obtained using a voltage ramp protocol. Recordings were done using the voltage ramp protocol depicted in (A). The holding potential was -40 mV. A voltage ramp of 300 ms duration from -120 mV to +60 mV was applied. Currents recorded from a TbIRK-expressing oocyte in sodium medium (NaME) and potassium medium (KME) are shown in (B) and (C). The I/V-relationship is very similar to the one obtained with the voltage-step protocol.

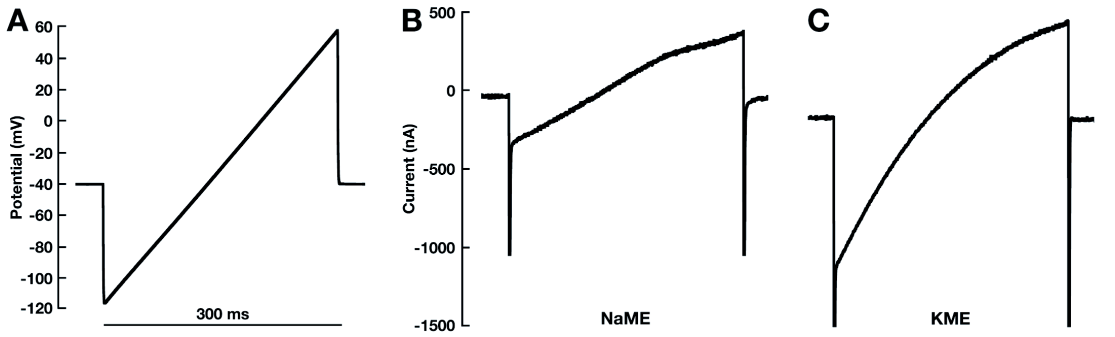

Figure S4. Averaged current-voltage relationship of oocytes expressing the TbIRK G132A mutant. All recordings were done by applying the voltage-step protocol depicted in Figure 2E. The current-voltage relationship in sodium medium (squares), potassium medium (closed circles) and potassium medium supplemented with 10 mM CsCl (open circles), respectively, were averaged (mean ± S.E.M.; n = 6).

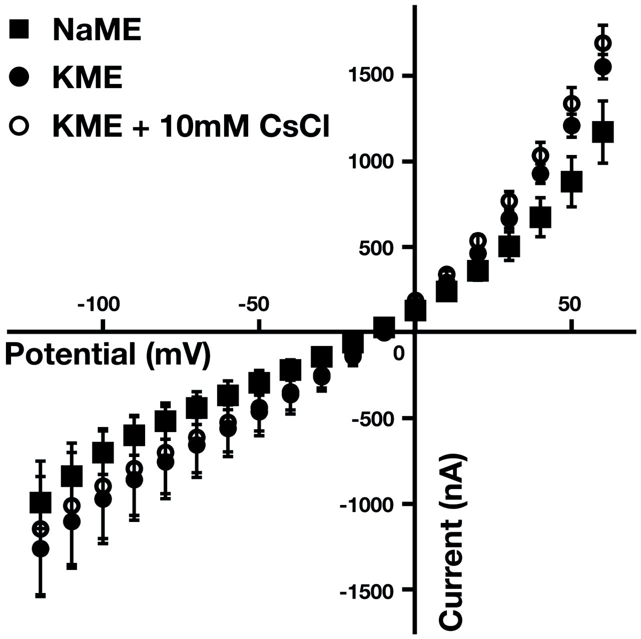

Figure S5. Western blot of HA-tagged versions of TbIRK. Induced (+) and non-induced (-) cell lines generated for localization experiments were analyzed by Western blot to confirm overexpression of the HA-tagged TbIRK. $5x10^6$ cells of procyclic form parasites (PCF) and $3x10^6$ cells of blood stream form parasites (BSF) were subjected to 8% SDS-polyacrylamide gel electrophoresis, transferred to nitrocellulose membranes, and probed with mouse anti-HA antibody. Expression of the tagged TbIRK could be confirmed (expected size: 63 kDa; lower bands). As loading control the membranes were re-probed with rabbit anti-BiP antibody (expected size: 75 kDa; upper bands). Relative position of the protein-ladder is shown on the left side. CHA: C-terminal HA-tag; NHA: N-terminal HA-tag.

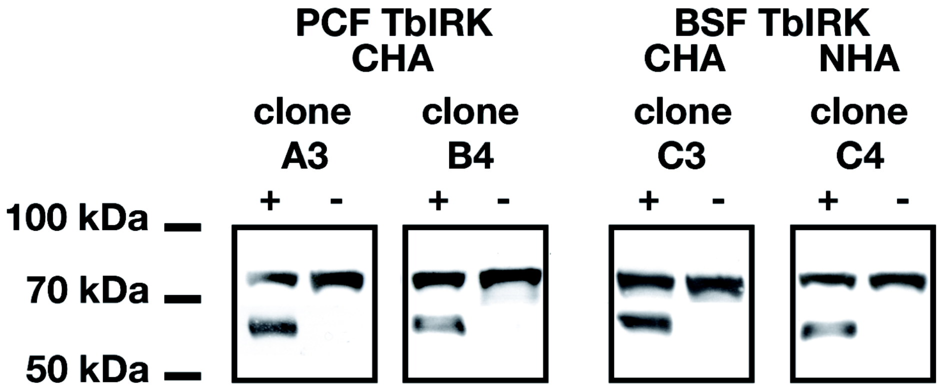

Table S1. Effect of known K_{ir}-channel blockers on TbIRK.

Compound	Concentration	Inhibition
Carvedilol	300 µM	n.s.
δ-Dendrotoxin	100 nM	n.s.
Lq2	10 nM	n.s.
Rosiglitazone	400 µM	39 ± 7 %
Tertiapin	100 nM	n.s.
Tertiapin-LQ	200 nM	n.s.
Tertiapin-Q	200 nM	n.s.
VU590 dihydrochloride	50 µM	n.s.
VU591 hydrochloride	50 µM	n.s.

Measurements were done using the voltage-step protocol depicted in Figure 2E. Inhibition was calculated from the current amplitude observed at -110 mV in potassium medium. Each potential inhibitor was tested 3-5 times on independent oocytes. n.s.: statistically not significant ($p > 0.05$).
